# Occurrence, Ecological Risk, and Human Exposure of Rubber Additives and Transformation Products in Surface Waters of Kaifeng, China

**DOI:** 10.3390/toxics14060521

**Published:** 2026-06-15

**Authors:** Xing Chen, Chenyang Sun, Lingnan Du, Xinding Yao, Haifeng Wang, Zongwu Wang, Jiapu Ji, Jinting Huang

**Affiliations:** 1 Henan Engineering Technology Research Center of Green Coating Materials, Kaifeng Engineering Technology Research Center of Aquatic Environmental Pollution Monitoring, Kaifeng Key Laboratory of Food Composition and Quality Assessment, School of Environmental Engineering, Yellow River Conservancy Technical University, Kaifeng 475004, China; nikinew@126.com (L.D.); yaoxinding126@126.com (X.Y.); wanghaifeng@yrcti.edu.cn (H.W.); kf0986@163.com (Z.W.); jjp9122005@163.com (J.J.); 2 Research and Development Center for Watershed Environmental Eco-Engineering, Advanced Institute of Natural Sciences, Beijing Normal University, Zhuhai 519087, China; 15893863095@163.com; 3 College of Surveying and Mapping Engineering, Yellow River Conservancy Technical University, Kaifeng 475004, China

**Keywords:** rubber additives, transformation products, 6PPD-Q, surface water, multi-pathway exposure, risk assessment

## Abstract

This study investigated rubber additives and relevant transformation products (RARTPs) in surface waters of Kaifeng, a city linking the Yellow River and Huaihe River basins. Seven of fifteen target analytes were detected in >10% of samples. The hydrolysis product 4-hydroxydiphenylamine (4OH) showed the highest detection frequency (70%), followed by 1,2-Dihydro-2,2,4-trimethylquinoline (TMQ, 57%) and *N*-(1,3-Dimethylbutyl)-*N*′-phenyl-*p*-phenylenediamine (6PPD, 27%). TMQ had the highest average concentration (6.16 ± 4.17 ng·L^−1^). Urban rivers (14.20 ± 4.72 ng·L^−1^) were contamination hotspots, driven by management practices (e.g., dredging of urban lakes). Although detected at lower levels (0.09 ± 0.21 ng·L^−1^), 6PPD-quinone (6PPD-Q) was associated with elevated risk (risk quotient, *RQ* ≥ 1) at 19% of sites. The chronic daily intake assessment showed that drinking water ingestion contributed 66.7% of total exposure in daily use, whereas dermal absorption dominated during swimming. Children, especially girls, were the most vulnerable subgroup. Although estimated chronic daily intakes (*CDI*s) from surface water accounted for only a negligible proportion of the daily urinary excretion of *p*-phenylenediamine antioxidants (PPDs) reported in a Chinese population, the ecological risk of 6PPD-Q warrants continued attention. These findings highlight the need for improved management of water bodies receiving urban runoff and aquaculture inputs, and further health risk assessment of RARTPs.

## 1. Introduction

Rubber additives constitute a significant category of synthetic organic chemicals, widely employed in the production of rubber products—particularly tires—as antioxidants, vulcanization inhibitors, and bonding agents [1,2]. As the world’s leading producer, China’s rubber chemical production reached approximately 1.239 million metric tons in 2020, accounting for nearly 75% of global output, with a year-on-year increase of 0.65% [3]. Tire wear is a major emission pathway, with global annual per capita emissions estimated between 0.2 and 5.5 kg [4]. The widespread use of rubber additives in tires, coupled with the ubiquitous presence of tire wear particles, leads to the continuous release of these substances into environmental media [5]. Among them, *p*-phenylenediamines (PPDs) represent one of the most important classes, with 6PPD being the most widely used antioxidant in tires. Another important group, the non-*p*-phenylenediamine antioxidants (NON-PPDs), includes TMQ, which provides long-term thermal–oxidative protection and is frequently detected in the environment. Therefore, water bodies that receive road runoff, snowmelt, and atmospheric deposition have become the primary transport pathways and main collection sites for these compounds [6,7]. There are particular concerns about the ecological hazards posed by certain rubber additives. For instance, 6PPD was found to be highly toxic to aquatic organisms, with median lethal concentrations (*LC*_50_) of 162 μg·L^−1^ for larvae of the native Chinese species *Gobiocypris rarus* and 443 μg·L^−1^ for zebrafish [8,9,10]. Under environmental conditions, 6PPD can undergo specific transformation processes, leading to the formation of various degradation products with distinct environmental behaviors and toxicities. Under aerobic conditions, 6PPD reacts with ozone to produce 6PPD-quinone (6PPD-Q), a transformation product with significantly enhanced toxicity. Its *LC*_50_ for coho salmon reaches as high as 0.095 μg·L^−1^ [11], and this oxidation process has been demonstrated to substantially exacerbate the environmental risks posed by road runoff and atmospheric deposition to surface water bodies. In aqueous environments, 6PPD can also undergo hydrolysis, yielding several transformation products, among which 4-hydroxydiphenylamine (4OH) and 4-nitrosodiphenylamine (4-NOH) are notable examples.

To date, research on rubber additives and their relevant transformation products (RARTPs) has largely emphasized their contamination levels in various environmental media, e.g., atmospheric particles, dust, surface/groundwater, runoff, snow, drinking water, wastewater, sediment, soil, and even some animal-derived food products (egg, honey, and fish) [12,13,14,15,16,17,18,19], and the internal exposure amount of RARTPs among humans [19,20,21,22]. However, there remains a significant knowledge gap regarding the influence of heterogeneous land use and the inherent characteristics of water bodies on the occurrence and fate of RARTPs in surface water systems. Specifically, there is a lack of systematic research examining variations in the pollution traits and sources of RARTPs across water bodies exposed to vastly different anthropogenic pressures (e.g., urban runoff and aquaculture inputs). Understanding these spatial and functional determinants is essential to accurately assess the transport mechanisms and ecological risks of RARTPs at the watershed scale. In practice, there is a need for guidance and a reference for surface water safety.

Kaifeng (a city in northern China) is often referred to as the “Water City of the North.” Although it is geographically located within the Huaihe River basin, the vast majority of its water resources (surface water and groundwater) still originate from the Yellow River via irrigation canals and lateral seepage. Therefore, the water network in this region is characterized by the combined use of surface water and groundwater for a variety of purposes, including drinking water supply, aquaculture, landscape and recreational uses, and storm water drainage. This unique setting provides an ideal model system for investigating the effects of diverse water sources, land use types, and management practices on the occurrence and fate of RARTPs in surface waters. The objectives of this study were: (i) to characterize the occurrence and spatial distribution of 15 RARTP contaminants in the surface water in Kaifeng City, (ii) to evaluate the potential daily intake of these contaminants through multiple exposure pathways to provide guidance on drinking water safety, and (iii) to conduct an in-depth investigation into the effects of specific land use types, management measures, and physicochemical properties on the migration, transformation, and fate of RARTPs in surface water.

## 2. Materials and Methods

### 2.1. Sample Collection

Kaifeng is located on the alluvial plain in the middle and lower reaches of the Yellow River, featuring a flat and open terrain. Over the course of the city’s long history within Chinese civilization and natural sedimentation processes, a thick silt layer has gradually accumulated, interbedded with various soil types and alluvial deposits. Due to the Yellow River’s typical wandering behavior, the subsurface stratum consists primarily of silt, fine sand, sand, and light loam, resulting in high permeability but relatively low mechanical strength. The climate of Kaifeng is warm, continental, and semi-arid monsoon, with clearly defined seasons. The multi-year average annual temperature ranges from 13.1 °C to 14.5 °C, and the average annual precipitation is approximately 600 mm, mostly falling between June and September. Historical average relative humidity varies between 65% and 75%. Elevation gradually decreases from northwest to southeast. According to the Kaifeng Statistical Yearbook 2023 [23], the city had a population of 4.69 million and a population density of 751 persons per square kilometer in 2023. The total number of automobiles in Kaifeng reached 830,000 in the same year, a 4.0% increase from the previous year’s end.

**Figure 1:**
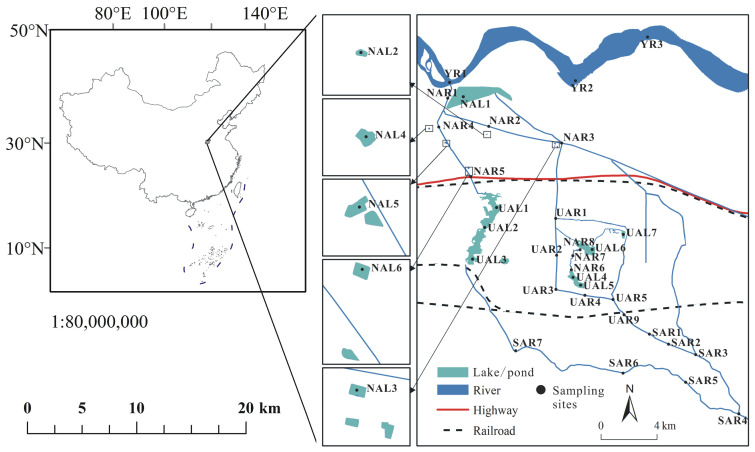
Map of sampling sites in Kaifeng, China.

As shown in Figure 1, the sampling sites start from the south bank of the Yellow River and are divided from north to south into the “Yellow River” (YR), “northern area rivers” (NARs), “northern area lakes” (NALs), “urban area rivers” (UARs), “urban area lakes” (UALs), and “southern area rivers” (SARs) (see Supplementary Material Table S1 for details). A total of 37 surface water samples were collected on 23 and 24 August 2023. Among them, samples YR1, YR2, YR3, NAR1, and NAL1 were collected from drinking water sources.

Two liters of surface water was collected as a sample at each sampling site using polyethylene bottles. These bottles were pre-rinsed in the laboratory with tap water, methanol, and Milli-Q water, and then rinsed three times with surface water at the sampling sites. Each composite sample consists of three samples collected sequentially at the same sampling site (approximately 1 m below the water surface) and thoroughly mixed afterward. Water samples were transported to the laboratory at 4 °C. The analysis of all collected water samples was conducted within two days after collection.

### 2.2. Analytical Methods

Basic information of the target analytes, reagents, and standards used in this study, including their CAS registry numbers, molecular formulae, purity, suppliers, and key physicochemical properties, is summarized in Table S2.

#### 2.2.1. Analysis of Surface Water Physiochemical Parameters

Total nitrogen (TN) and total organic carbon (TOC) are two of the physical characteristics of surface water. A total organic carbon analyzer (TOC-L CPH, SHIMADZU, Kyoto, Japan) was used to measure the TOC and TN contents in surface water samples. TOC was computed using the formula that states that TOC is equal to the total carbon (TC) amount minus the inorganic carbon (IC) amount.

#### 2.2.2. Sample Extraction and Instrumental Analysis for RARTPs

1. Sample extraction

The process for extracting and instrumentally analyzing RARTPs is based on a previously published method [24,25]. In brief, for each 300 mL surface water sample, filtration was performed using a 0.7-micron GF (glass microfiber filter)/F membrane (Hangzhou Special Paper Industry Corp., Ltd., Hangzhou, China), and these filters were pre-cleaned for optimal filtration performance. The filtrates were subsequently extracted using an automatic high-flux solid-phase extractor (Fotector Plus, RayKol Group Corp., Ltd., Xiamen, China) coupled with Oasis hydrophilic–lipophilic balance solid-phase extraction cartridges (500 mg·6 mL^−1^, Waters, Milford, MA, USA).

2. Instrumental analysis

For each cartridge, pre-treatment was performed sequentially with 10 mL of methanol followed by 10 mL of deionized water. Subsequently, the filtrate was injected into the cartridge at a flow rate of approximately 1.5 mL·min^−1^. Then, the cartridges were washed using 10 mL of mixed solvent (equal volumes of methanol and deionized water) and left to dry under nitrogen for 30 min. After drying, 15 mL of methanol and 10 mL of acetonitrile were used to elute the analytes from the HLB cartridges. The eluate was ultimately concentrated to 0.5 mL and diluted with deionized water to a final volume of 1 mL for analysis via ultra-high-performance liquid chromatography and mass spectrometry (UHPLC-MS/MS).

The analysis included seven *p*-phenylenediamine antioxidants (PPDs), namely 6PPD, 7PPD, 8PPD, CPPD, DNPD, DPPD, and IPPD, two *p*-phenylenediamine quinones (PPD-Qs), namely 6PPD-Q and IPPD-Q, two hydrolysis products (HPs), namely 4-NOH and 4OH, three non-*p*-phenylenediamine antioxidants (NON-PPDs), including 445, 2N, and TMQ, one binding agent (BA), namely HMMM, and was conducted utilizing an Agilent 1290 Infinity II ultra-high-performance liquid chromatograph equipped with ZORBAX Eclipse Plus C18 column (Rapid Resolution HD 2.1 × 100 mm, 1.8-micron), paired with an Agilent 6470 triple quadrupole mass spectrometer (UHPLC-MS/MS, Agilent Technologies Inc., Santa Clara, CA, USA). The injection volume is 2 μL. The mass spectrometer is equipped with an Agilent jet stream electrospray ionization (AJS ESI) probe, which measures all target compounds in positive ion mode. Methanol (A) and water containing 0.1% formic acid (B) served as the mobile phase, which was pumped at a flow rate of 0.3 mL·min^−1^. The gradient varied as follows: 0 min, 50% A; 3 min, 98% A; 8 min, 98% A; 8.1 min, 50% A; and 10 min, 50% A. The identification and quantification of all analytes were conducted using multiple reaction monitoring (MRM) mode. The complete MS method details, including precursor/product ions, retention time, collision energy, fragmentor, detection limits, and calibration curve linearity, are elucidated in Table 1 and Table S3.

3. Quality Control/Quality Assurance

To minimize background errors in quality control/quality assurance, regular testing was conducted on blank samples and matrix samples using the standard addition method, sample replicates, method blanks, and field blanks. The recovery rates for these analytes all reached satisfactory levels, ranging from 52.7% to 115.6%. For each set of twenty samples, duplicate blank samples were analyzed to evaluate the extraction and analytical procedures. The relative variation for duplicate samples in each batch was consistently below 9.3%.

### 2.3. Risk Assessment

#### 2.3.1. Ecological Risk Assessment for Surface Water Phase

For water samples, the *LC*_50_ method (i.e., the concentration causing 50% mortality in three representative aquatic organisms—algae, invertebrates, and fish) was employed to assess the ecological risk of RARTPs [18,26,27] (see Supplementary Material Text S1 for equations and risk categories).

#### 2.3.2. Exposure Assessment of Chronic Daily Intakes (*CDI*s)

To describe the exposure of RARTPs (PPDs, PPD-Qs, HPs, NON-PPDs, and BA), the *CDI* assessment of RARTPs in surface water samples was performed following the models recommended by the U.S. Environmental Protection Agency [28,29]. This study emphasized the assessment of *CDI*s for RARTPs in surface water under three exposure scenarios. Scenario 1 considers exposure resulting from drinking and bathing with tap water sourced from drinking water supplies; Scenario 2 assesses exposure from swimming in other surface water bodies (in accordance with local customs); and Scenario 3 involves exposure resulting from a combination of daily use (drinking and bathing) and swimming in other surface water bodies (equations, variables, and parameters are given in Supplementary Materials) [30,31,32,33,34,35,36].

## 3. Results and Discussion

### 3.1. RARTPs in the Surface Water of the Study Area

**Figure 2:**
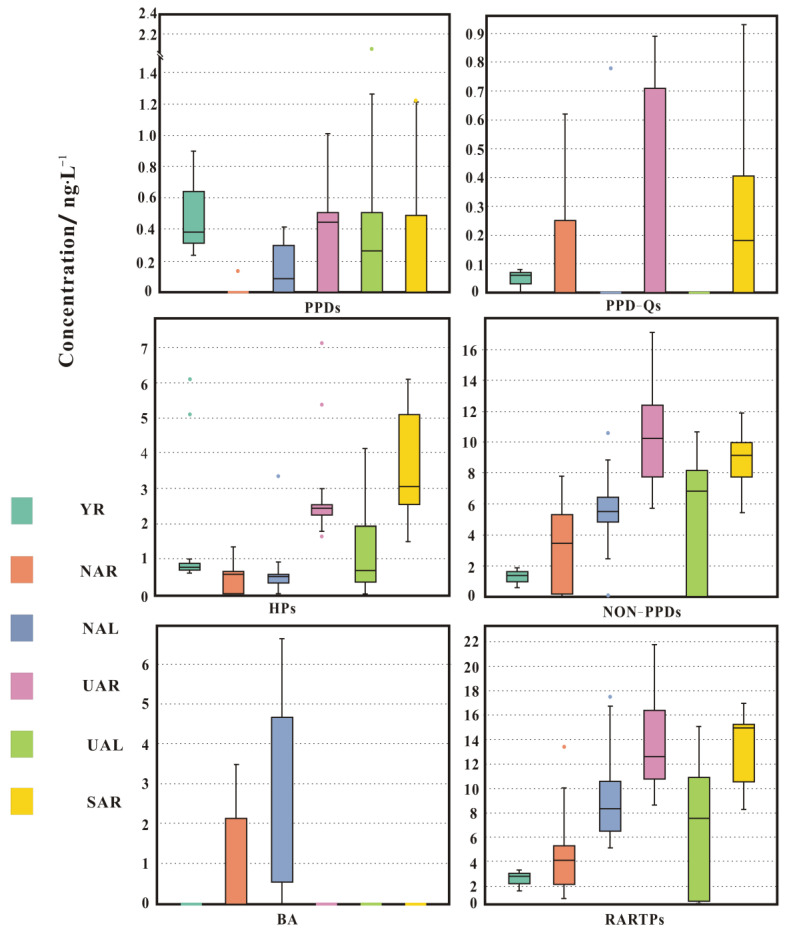
Box plots showing the concentrations of RARTPs in surface water from all sampling locations (YR: the Yellow River, NAR: northern area river, NAL: northern area lake, UAR: urban area river, UAL: urban area lake, SAR: southern area river).

The spatial distributions and descriptive statistics for the concentrations of RARTPs in surface water samples are shown in Figure 2 and Table 2. For the study area, five of the 15 analytes were not detected (ND) (8PPD, CPPD, DNPD, DPPD, IPPD, and 445), and three were detected in no more than 10% of the samples (7PPD, 6PPD-Q, and 4-NOH), with the range of 5.41–8.11%. In over 10% of surface water samples, seven targets were found, with 4OH (hydrolysis product of 6PPD) identified as the most dominant congener, with the highest detection frequency of 70%, followed by NON-PPDs, TMQ (57%), 2N (27%) and the antioxidant 6PPD (27%). For RARTPs, the concentrations of TMQ (6.16 ± 4.17 ng·L^−1^, range: ND–16.52 ng·L^−1^), followed by 4OH (1.92 ± 1.82 ng·L^−1^, range: ND–7.14 ng·L^−1^) and HMMM (0.60 ± 1.55 ng·L^−1^, range: ND–6.63 ng·L^−1^), were among the highest. As the representative PPD, 6PPD ranged from ND to 1.64 ng·L^−1^. When compared with PPDs, PPD-Qs, and a BA, the concentrations of NON-PPDs and HPs were nearly an order of magnitude greater (Table 2).

The spatial distribution of RARTPs associated with enhanced human activity (Figure 2 and Table 3) reveals a distinct north-to-south gradient of pollutant concentrations within the river systems. Concentration levels were as follows: Yellow River (YR) (2.59 ± 0.88 ng·L^−1^) < northern area rivers (NARs) (5.16 ± 4.88 ng·L^−1^) < urban area rivers (UARs) (14.20 ± 4.72 ng·L^−1^), with the latter concentration approaching that of southern area rivers (SARs) (13.10 ± 3.26 ng·L^−1^). Additionally, significant differences exist in pollutant concentrations between rivers and lakes (or ponds) within the same region. On average, pollutant concentrations in northern area rivers are lower than those in northern area lakes (or ponds) (9.41 ± 4.49 ng·L^−1^). In contrast, the opposite trend is observed in urban areas, where pollutant concentrations in urban area rivers (UARs) are higher than in urban area lakes (UALs) (6.64 ± 6.21 ng·L^−1^). Thus, urban rivers represent critical hotspots for RARTP contamination.

Limited information is available on the occurrence of RARTPs in surface water. Table 4 and Table 5 review contents of 6PPD, IPPD, and 6PPD-Q detected in various water samples in China [18] and other countries [2,5,14,37,38,39]. The contents of 6PPD in surface water were higher in Kaifeng (ND–1.64 ng·L^−1^, 0.20 ± 0.38 ng·L^−1^) than those reported for the Liuxi River in Guangzhou (China) and for the stormwater from Don River and Highland Creek (Canada), but were markedly lower than the runoff concentrations in Saskatoon (Canada) (86–1400 ng·L^−1^) and the wastewater effluent in Leipzig (Germany) (ND–105 ng·L^−1^). The IPPD concentrations were ND–0.46 ng·L^−1^ (0.07 ± 0.14 ng·L^−1^), which were slightly lower than the surface water of Liuxi River, China (avg: 0.31 ng·L^−1^). The content of 6PPD-Q in the surface water in Kaifeng was slightly lower than that of surface water in the Liuxi River, and was much lower than those in various waters reported elsewhere. In addition, compared with the concentrations from existing sampling sites of urban surface water across major river basins in China (provincial capital and another major city) [40], the PPD levels in Kaifeng surface water were slightly lower than those in the Yellow River basin, and substantially lower than those in the Hai River, Yangtze River, and Pearl River basins. Overall, the contamination levels of RARTPs in Kaifeng surface water were relatively low, with the concentrations of the high-risk PPD-Qs being substantially lower than those reported in other major river basins of China and in international studies. However, it should be noted that the monitoring data in this study were collected exclusively during the summer season. Considering the strong seasonal variability in PPD-Q photochemistry and runoff events, future multi-seasonal monitoring is warranted to validate these findings.

In comparison with the concentrations of RARTPs documented in our prior study on the groundwater within the research area (Table 2) [24], the surface water pollutant levels of PPDs (ND–2.05 ng·L^−1^, 0.31 ± 0.44 ng·L^−1^), PPD-Qs (ND–0.93 ng·L^−1^, 0.18 ± 0.31 ng·L^−1^), and BA (ND–6.63 ng·L^−1^, 0.60 ± 1.55 ng·L^−1^) were lower than those of groundwater (PPDs, ND–44.63 ng·L^−1^, avg: 9.59 ng·L^−1^, PPD-Qs, ND–4.65 ng·L^−1^, avg: 0.72 ng·L^−1^, BA, ND–11.05 ng·L^−1^, avg: 1.59 ng·L^−1^). However, the content of NON-PPDs (ND–17.09 ng·L^−1^, 6.54 ± 4.40 ng·L^−1^) and HPs (ND–7.14 ng·L^−1^, 1.99 ± 1.89 ng·L^−1^) in the surface water was slightly higher than that of groundwater (ND).

Compared with the general pollutants in surface water reported in previous research, the pollutant levels of RARTPs were slightly lower than those of antibiotics (a few to hundreds of ng·L^−1^) [41,42], lower than those of short-chain chlorinated paraffins and perfluoroalkyl substances (dozens to thousands of ng·L^−1^) [43,44,45], polybrominated diphenyl ethers [46,47], neonicotinoid insecticides and PAHs (hundreds to thousands of ng·L^−1^) [48,49], and significantly lower than those of heavy metals (up to hundreds of mg·L^−1^) [50].

Differences in water sources, land use, and management practices can explain the spatial disparity in RARTP concentrations across water bodies. In the northern suburban area, fishponds are supplied primarily with local groundwater, which inherently contains elevated levels of the target pollutants. This leads to an increased accumulation of pollutants in fishponds. In contrast, the northern rivers serve as diversion channels, carrying water from the Yellow River, which is a source with relatively low pollutant loads. This results in markedly lower concentrations. The stark contrast between rivers and lakes in the city center reflects the impact of management measures. Urban lakes, which are maintained as landscape features, undergo regular dredging. This effectively removes historical pollutants and reduces pollutant concentrations in the water. In addition, the lower pollutant levels in urban lakes could also be explained by longer water residence times promoting degradation and dilution by cleaner water inputs. On the contrary, urban rivers continuously receive surface runoff and municipal wastewater discharges, thus exhibiting higher concentrations of pollutants. Overall, these findings show that the quality of source water and distinct management practices are key factors in determining the spatial distribution of RARTPs within heterogeneous urban water systems.

Due to the significant differences in environmental geochemical composition between surface water and groundwater, the concentrations of precursor chemicals (PPDs) and their oxidation products (PPD-Qs) are lower in surface water. Consequently, the levels of their hydrolysis products (HPs) are higher in surface water than in groundwater. This disparity in fate may stem from the eutrophication of the surface water. During summer sampling periods, algal blooms proliferate intensively and release colored dissolved organic matter, imparting a distinctive greenish hue to the water body and significantly altering its optical properties [51]. Phytoplankton pigment deposits significantly reduce the penetration of solar radiation by absorbing ultraviolet and visible light radiation (<280 nm) [52]. This shielding effect has been demonstrated to have a direct impact on photochemical pathways. In natural water bodies, the photolysis of organic pollutants primarily occurs indirectly through a process mediated by reactive species generated by ultraviolet irradiation [8,53,54]. Consequently, the oxidation of PPD into PPD-Qs is inhibited by the attenuation of UV light. Conversely, the hydrolysis pathway that results in the production of HPs is a non-photochemical, non-biological process that functions independently of light exposure conditions. High amounts of HPs accumulate as a result of hydrolysis becoming a comparatively more dominant process in shadowed surface water habitats. In contrast to the clearer groundwater environment, inhibited photochemical oxidation leads to a decrease in the formation of PPD-Qs.

### 3.2. Factors Controlling Pollution Patterns in Surface Water RARTPs

**Figure 3:**
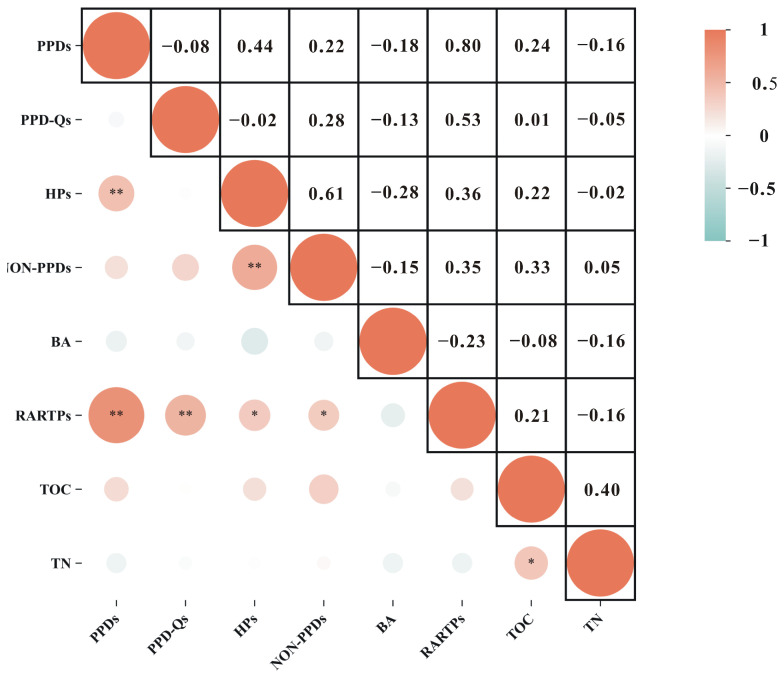
Spearman’s correlations of PPDs, PPD-Qs, HPs, NON-PPDs, BA, total RARTPs, TOC, and TN; The color intensity and size of the circles are proportional to the absolute *r* value; ** correlation is significant at the 0.01 level; * correlation is significant at the 0.05 level.

Figure 3 displays the Spearman correlations between PPDs, PPD-Qs, HPs, NON-PPDs, BA, total RARTPs, TOC, and TN. Within surface water throughout the research region, no significant correlation was found between PPDs and PPD-Qs (*r* = −0.08, *p* = 0.63), while a significant positive correlation was observed between PPDs and HPs (*r* = 0.44, *p* < 0.01). This supports the earlier conclusion that eutrophication-induced UV attenuation inhibits the photochemical conversion of PPDs to PPD-Qs, making hydrolysis a relatively more important pathway for HP formation in surface water.

No significant relationship was observed between the contents of PPDs and TOC (*r* = 0.24, *p* = 0.17) or TN (*r* = −0.16, *p* = 0.36), indicating that the presence of nutrient element TN did not significantly enhance the accumulation of highly toxic RARTPs. Previous studies indicated that TOC has essential implications in controlling the fate and behaviors of PAHs in soils and sediments [48,55]. Nevertheless, the amount of PPDs in the surface water of the study area was not primarily determined by TOC. This supports the earlier study [56] which found no correlation between TOC and the contents of sediment PPDs. As a result, one potential mechanism impacting PPD distribution could be the transformation of PPDs in natural environmental conditions.

The present study is constrained by a limited assessment of water physicochemical properties. The absence of key parameters (e.g., major ions, total Dissolved Solids, pH, oxidation-reduction potential, dissolved oxygen, conductivity, and turbidity) restricts a full elucidation of the hydrological and geochemical mechanisms driving RARTP behavior, as these factors may influence the speciation and transformation of organic pollutants [57,58]. To ensure a robust evaluation of the environmental fate and generalizability of RARTPs, future research must incorporate these essential physicochemical indicators alongside TN and TOC.

### 3.3. Ecological Risk Assessment

**Figure 4:**
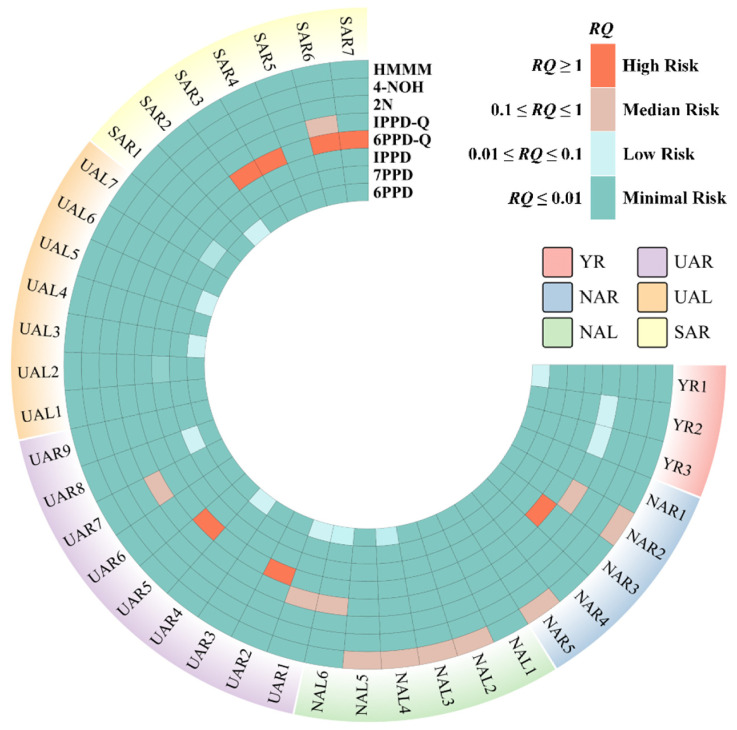
Distribution of risk quotients of target RARTP pollutants (6PPD, 7PPD, IPPD, 6PPD-Q, IPPD-Q, 2N, 4-NOH, and HMMM) in surface water samples.

The ecological risks of RARTPs with a detection rate greater than 0 percent in surface water in the study area were evaluated according to Equation (S1) (6PPD, 7PPD, IPPD, 6PPD-Q, IPPD-Q, 2N, 4-NOH, and HMMM), and the risk quotients (*RQ*s) for each site are shown in Figure 4. For the surface water of the study area, ecological risk caused by IPPD, 2N, and 4-NOH could be ignored (minimal risk, *RQ* < 0.01). In total, 16%, 3%, and 5% of the samples indicated a low risk of 6PPD, 7PPD, and IPPD-Q (0.01 ≤ *RQ* < 0.1). Additionally, 16% and 14% of the samples showed a median risk of HMMM and IPPD-Q (0.1 ≤ *RQ* < 1), respectively. Having the highest *RQ* value of 8.79, 6PPD-Q revealed high ecological risks (*RQ* ≥ 1) in 19% of the sampling sites. These findings are consistent with former research [18] that compared 6PPD-Q with other chemical additions or transformation products; 6PPD-Q was thought to be the primary cause of RARTP-mediated aquatic toxicity in source water. Thus, greater consideration should be given to 6PPD and its oxidation product, 6PPD-Q, since the high-risk probability of 6PPD-Q may result in greater toxicity even at lower concentrations. However, it is worth noting that the pronounced eutrophication observed in surface water bodies during the summer months may inhibit the photochemical oxidation of PPDs to PPD-Qs, thereby partially mitigating the potential aquatic risks posed by PPD-Qs under actual field conditions. Additionally, it has been reported that chronic exposure to environmentally relevant concentrations of 6PPD-Q might result in intestinal toxicity in organisms by interfering with the intestinal barrier’s ability to function [59]. Given that surface water in the study area could be used for irrigation, aquaculture, daily drinking, cleaning, etc., future studies should continue to focus on the potential environmental risks that could arise from RARTP contamination attribution in subsequent surface water use scenarios.

### 3.4. Exposure Assessment (Chronic Daily Intakes, CDIs)

**Figure 5:**
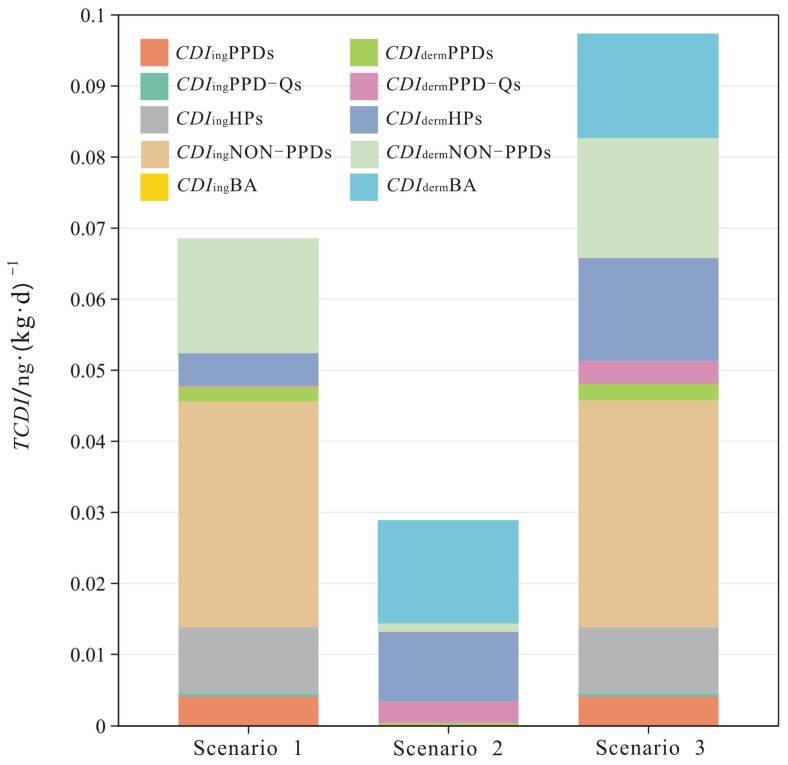
Scenario-specific *CDI*s of local residents exposed to RARTPs from surface water in the study area (for Scenario 1, *CDI*_ing_ represents *CDI*_ing-drink_, and *CDI*_derm_ represents *CDI*_derm-bath_; for Scenario 2, *CDI*_ing_ represents *CDI*_ing-swim_, and *CDI*_derm_ represents *CDI*_derm-swim_; for Scenario 3, *CDI*_ing_ represents the combination of *CDI*_ing-drink_ and *CDI*_ing-swim_, and *CDI*_derm_ represents *CDI*_derm-bath_ and *CDI*_derm-swim_).

**Figure 6:**
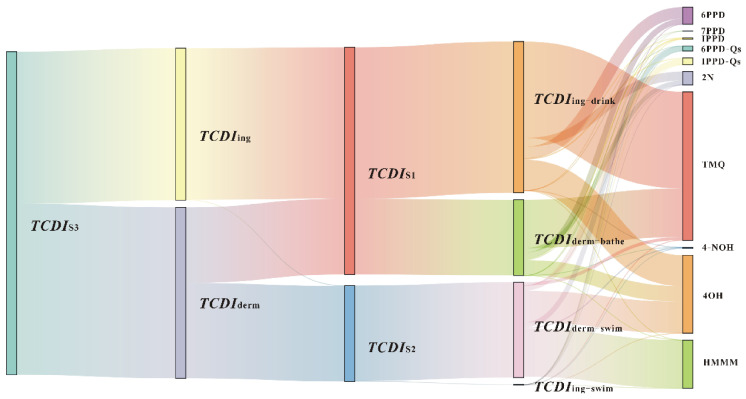
Relative contribution of individual RARTPs and exposure pathways to scenario-specific *TCDI* values for local residents.

Equations (S2)–(S5) were applied to calculate the chronic daily intake (*CDI*_ing_, *CDI*_derm_, and total chronic daily intake, *TCDI*) of RARTP pollutants (PPDs, PPD-Qs, HPs, NON-PPDs, and BA) in all surface waters for the local population (including different genders and age groups) under three exposure scenarios. As shown in Figure 5 and Figure 6, the *TCDI* assessment results for Scenarios 1, 2, and 3 were 6.84 × 10^−2^ ng·(kg·d)^−1^, 2.88 × 10^−2^ ng·(kg·d)^−1^, and 9.73 × 10^−3^ ng·(kg·d)^−1^, respectively.

Regarding exposure pathways, in Scenario 1 (daily water consumption), *CDI*_ing-drink_ is 4.56 × 10^−2^ ng·(kg·d)^−1^ and *CDI*_derm-bathe_ is 2.28 × 10^−2^ ng·(kg·d)^−1^, with *CDI*_ing-drink_ accounting for 66.7% of *TCDI*. Meanwhile, the *CDI*_ing-swim_ value in Scenario 2 was only 1.40 × 10^−4^ ng·(kg·d)^−1^, while dermal intake (*CDI*_derm-swim_) was 2.87 × 10^−2^ ng·(kg·d)^−1^, implying that dermal intake accounts for only 0.5% of the total. Variations in exposure frequency between the two scenarios contributed to this significant difference. Specifically, under swimming conditions, both the unintentional water ingestion rate (*IR*_swim_) and the exposure frequency for swimming (*EF*_swim_) are significantly lower than those of daily drinking and bathing (*EF*_drink/bathe_) (Table S4). This results in a much lower intake dose during swimming, even if the concentration of pollutants in the source water is relatively lower than in other surface water bodies.

Under the scenario where only daily water consumption is considered (Scenario 1), the highest exposure levels for RARTP pollutants were observed for non-PPDs (4.77 × 10^−2^ ng·(kg·d)^−1^), followed by HPs (1.40 × 10^−2^ ng·(kg·d)^−1^) and PPDs (6.16 × 10^−3^ ng·(kg·d)^−1^). The concentration of NON-PPD compounds remained the highest in Scenario 3 (combination of daily use and swimming), at 4.88 × 10^−2^ ng·(kg·d)^−1^, followed by HP compounds (2.39 × 10^−2^ ng·(kg·d)^−1^), BA (1.45 × 10^−2^ ng·(kg·d)^−1^), PPDs (6.45 × 10^−3^ ng·(kg·d)^−1^), and PPD-Qs (3.60 × 10^−3^ ng·(kg·d)^−1^). Furthermore, across all RARTPs, *CDI*_TMQ_ contributed 46.0% to the *TCDI* value, followed by *CDI*_4OH_ (24.2%), *CDI*_HMMM_ (14.9%), and *CDI*_6PPD_ (5.5%) (Figure 6).

The daily excretion (*DE*) and the chronic daily intake of PPDs in human urine were compared to comprehensively assess the exposure levels of the general population to RARTPs. In a study conducted in eastern China in 2019, the *DE* value of this population was 87.2 ng·(kg·d)^−1^ [22], whereas the average chronic daily intake (*CDI*) of PPDs from surface water in the study area was 5.37 × 10^−3^ ng·(kg·d)^−1^, accounting for only 0.01% of the population’s *DE*. Thus, for the local population, other environmental media (e.g., atmospheric particles, dust, and food) may play a more significant role in chronic RARTP exposure than surface water.

A Monte Carlo simulation was run 10,000 times to obtain the cumulative probability distributions of the computed *TCDI* for each population group under Scenario 3 (Table S5). Subgroup analyses revealed different patterns of chronic daily intake, with *TCDI* values showing a downward trend: girl (50th: 1.57 × 10^−1^ ng·(kg·d)^−1^) > boy (50th: 1.42 × 10^−1^ ng·(kg·d)^−1^) > male adult (50th: 1.16 × 10^−1^ ng·(kg·d)^−1^) > female adult (50th: 1.10 × 10^−1^ ng·(kg·d)^−1^). For the local adult population, the average weights were 38.0 kg (boys), 34.9 kg (girls), 66.6 kg (men), and 57.9 kg (women) [31,32,33,35]. The central tendency exposure (50th) values of the daily RARTP intake were 5.97, 4.96, 7.73, and 6.37 ng·d^−1^, and reasonable maximum exposure (90th) values were 7.49, 5.72, 14.19, and 8.80 ng·d^−1^, respectively.

It was indicated by the ecological risk assessment (Section 3.3) that a high ecological risk (*RQ* ≥ 1) is posed by 6PPD-Q at 19% of the sampling sites. Given the extremely low *LC*_50_ of 6PPD-Q (0.095 μg·L^−1^), concerns are raised about potential health risks to the local population from daily use and swimming in the surface water bodies within the study area. However, current toxicological knowledge regarding 6PPD-Q is largely derived from model organisms such as fish and zebrafish, with documented effects including acute lethality, developmental toxicity, and reproductive impairment, while direct evidence of specific adverse health outcomes in humans (particularly in children) remains lacking [60,61,62,63]. Unfortunately, it was not possible to conduct a human health risk assessment in this study due to the lack of key parameters such as the slope factor (*SF*) or the carcinogenic reference dose (*RfD*). Future investigations should therefore establish risk thresholds to develop a comprehensive understanding of the health risks associated with RARTP contamination in water.

Furthermore, it has been indicated in previous studies that children are a sensitive group with respect to environmental pollution, including heavy metals, PAHs, and disinfection by-products (DBPs) through drinking water, groundwater, food, suspended particles, dust, and soil [64,65,66,67,68,69]. Additionally, Chen et al. [65] found that females are more susceptible than males to the negative effects of DBPs in tap water throughout childhood, but this risk decreases with age. Their findings align with the results of this study, which show that *CDI* scores follow a decreasing trend across the following subgroups: girls > boys > adult men > adult women. Regardless of gender, the chronic daily intake of RARTPs from surface water is higher for children than for adults. Accordingly, the chronic daily intake profile for each subgroup may be significantly influenced by both the intake rate per kilogram of body weight and individual body weight. As a result, the potential health risks that children, especially girls, face from exposure to surface water RARTPs require more attention.

The present exposure assessment acknowledges certain limitations and uncertainties. Assuming direct surface water ingestion may not accurately represent the actual exposure pathways for local people as municipal tap water is prevalent for the study area. This study did not consider water treatment effects, where ozonation may transform PPDs into more toxic PPD-Qs, while activated carbon reduces overall loads. Future research should investigate these transformations and removal efficiencies to refine exposure risk assessments. In addition, from a health perspective, the Chinese population typically drinks boiled water, as untreated water is associated with greater health risks than boiled water [70]. Furthermore, a former study noted that there is a growing trend among Chinese residents to use filtered tap water or bottled/barreled water [24]. Given that the concentrations of RARTPs within different types of drinking water may exhibit variations, estimates of exposure levels are expected to fluctuate significantly.

In addition to drinking, swimming is another significant route of exposure, mainly through dermal contact and unintentional water ingestion. However, there are significant differences in exposure levels across different age and gender groups. A survey of Chinese children showed that the proportion of children engaged in swimming exhibits an age-related pattern of increase followed by a decrease, peaking at 21.7% among 9 to 11 year-olds; within this age group, engagement rates were generally higher among boys than girls. Furthermore, among children with swimming habits, the average duration of swimming sessions increases with age [33]. These demographic differences suggest that exposure assessments based on a single or generalized swimming scenario struggle to accurately reflect the true risks for various subgroups. Therefore, future studies should incorporate swimming frequency, duration, and accidental water ingestion rates specific to different ages and genders to improve the accuracy of RARTP exposure assessments.

Variability and uncertainty associated with surface water RARTP exposure pathways—which have the greatest impact on the estimated chronic daily intake—were assessed using sensitivity analysis. Figure S1 shows the percentile contribution to the variance for the sensitivity analysis results regarding *CDI*_ing_ and *CDI*_derm_. Regarding direct ingestion routes (*CDI*_ing-drink_ and *CDI*_ing-swim_), *C*_w_ was the primary determinant for both males and females, accounting for an average of 78% of the total variation in *CDI*_ing_. BW contributed −40% to *CDI*_ing-drink_, while *IR*_swim_ contributed 61% to *CDI*_ing-swim_. *C*_w_ remained the predominant influencing factor in both transdermal absorption pathways (*CDI*_derm-bathe_ and *CDI*_derm-swim_), accounting for an average of 69% of the variance. In terms of *CDI*_derm-bathe_, *BW* (for male, −65%) and *SA* (for female, 52%) also exhibited notable influences on the total variance. Based on the sensitivity analysis results, it is crucial to improve the accuracy of *C*_w_, given its consistent dominance in contributing to exposure variance across all pathways. Despite this, notable contributions from *IR*_swim_ and *SA* indicate that specific parameters related to recreational water activities should also be carefully identified.

## 4. Conclusions

The occurrence, spatial distribution, ecological risk, and chronic daily intake of RARTPs were systematically characterized in surface waters of Kaifeng, a major city adjacent to the Yellow River that serves as a hydrological link between the Yellow River and the Huaihe River basins. Consistent with the current production and use of rubber additives, 6PPD remained the predominant PPD, while its hydrolysis product 4OH and the NON-PPD antioxidant TMQ were detected at higher concentrations and frequencies. Across the study area, urban rivers stand out as the most significant pollution hotspots. Human activities, including urban runoff, municipal wastewater discharge, and aquaculture pond operation using diverted Yellow River water, were identified as key contributors to RARTP pollution. Management practices such as regular dredging of urban lakes effectively reduced pollutant levels, whereas urban rivers continuously received contamination from surface runoff and wastewater. The ecological risk caused by 6PPD-Q to surface water deserves attention (*RQ* ≥ 1 at 19% of sites). Exposure assessment revealed that ingestion of drinking water dominates daily exposure (66.7%), while dermal contact becomes more important under swimming scenarios. Children, especially girls, were the most vulnerable population due to their higher intake rate per kilogram of body weight. Although the estimated chronic daily intake from surface water accounted for only a minor fraction of the total internal exposure reported in a Chinese population, the high toxicity of 6PPD-Q and its widespread detection call for further research. Future studies should incorporate age- and sex-specific behaviors, and establish health-based reference doses to enable a comprehensive human health risk assessment of these unregulated contaminants in surface water.

## Figures and Tables

**Table 1:** Precursor and product ions ([M + H]^+^), retention time, collision energy, and fragmentor details of the target compounds for the multiple reaction monitoring mass spectrometer method.

	Compound Name	Retention Time (min)	Precursor Ion	Fragmentor	Product Ion 1	Collision Energy 1	Product Ion 2	Collision Energy 2
*p*-Phenylenediamine Antioxidants (PPDs)	6PPD	3.2	269.19	116	184.2	25	107.1	57
	7PPD	3.6	283.21	131	184.3	25	185	17
	8PPD	3.9	297.23	131	184.4	29	107	60
	CPPD	3.0	267.18	131	185	17	93.1	41
	DNPD	5.4	361.16	190	234.1	37	218.9	33
	DPPD	4.7	161.13	151	184.1	33	107.1	57
	IPPD	1.1	227.15	111	184.2	17	107.1	45
*p*-Phenylenediamine Quinones (PPD-Qs)	6PPD-Q	4.8	299.17	146	215	17	187.1	33
	IPPD-Q	3.8	257.22	136	187.1	29	77.1	60
Hydrolysis products (HPs)	4-NOH	3.6	199.08	65	181.2	29	128.1	49
	4OH	2.9	186.08	111	109	29	92.3	21
Non-p-Phenylenediamine Antioxidants (N-PPDs)	445	6.5	406.25	136	196.2	49	91.2	60
	2N	1.01	185.1	121	108	29	80.3	60
	TMQ	4.1	174.12	131	144.3	33	91.1	41
Binding Agent (BA)	HMMM	3.2	391.4	91	177.1	29	359.1	5
Surrogate Standard	6PPD-Q-d5	4.8	304.2	151	220.1	17	192.1	33
Internal Standard	BP-d10	4.1	193.22	106	82	37	109.9	17

**Table 2:** RARTP concentrations (ng·L^−1^) in surface/groundwater samples in the study area.

			Surface Water Samples (*n* = 37)					Groundwater Samples (*n* = 51) [24]
			DF *^a^*	Mean	SD	95th	Range	
Antioxidants	PPDs	6PPD	27.03%	0.20	0.38	1.22	ND–1.64	ND–42.96, avg: 4.05
		7PPD	8.11%	0.03	0.12	0.41	ND–0.58	ND
		8PPD	0%	ND *^b^*	-	ND	ND	ND
		CPPD	0%	ND	-	ND	ND	ND–4.45, avg: 0.68
		DNPD	0%	ND	-	ND	ND	ND
		DPPD	0%	ND	-	ND	ND	ND
		IPPD	21.62%	0.07	0.14	0.44	ND–0.46	ND–3.86, avg: 3.16
		ΣPPDs		0.31	0.44	1.22	ND–2.05	ND–44.63, avg: 9.59
	NON-PPDs	445	0%	ND	-	ND	ND	ND
		2N	27.03%	0.37	0.49	1.71	ND–1.78	ND
		TMQ	56.76%	6.16	4.17	13.24	ND–16.52	-
		ΣNON-PPDs		6.54	4.40	13.62	ND–17.09	ND
Binding agent	BA	HMMM	16.22%	0.60	1.55	5.33	ND–6.63	ND–11.05, avg: 1.59
Transformation products	PPD-Qs	6PPD-Q	8.11%	0.09	0.21	0.71	ND–0.84	ND–2.86, avg: 0.83
		IPPD-Q	16.22%	0.09	0.23	0.78	ND–0.89	ND–1.79, avg: 0.30
		ΣPPD-Qs		0.18	0.31	0.89	ND–0.93	ND–4.65, avg: 0.72
	HPs	4-NOH	5.41%	0.07	0.13	0.37	ND–0.40	ND
		4OH	70.27%	1.92	1.82	5.74	ND–7.14	-
		ΣHPs		1.99	1.89	6.10	ND–7.14	ND

*^a^* Detection frequency (%); *^b^* not detected.

**Table 3:** RARTP concentrations (ng·L^−1^) in surface water samples from different areas (*n* = 37).

	Northern Area (*n* = 14)									Urban Area (*n* = 16)						Southern Area (*n* = 7)		
	YR (*n* = 3)			NAR (*n* = 5)			NAL (*n* = 6)			UAR (*n* = 9)			UAL (*n* = 7)			SAR (*n* = 7)		
	Mean	SD	Range	Mean	SD	Range	Mean	SD	Range	Mean	SD	Range	Mean	SD	Range	Mean	SD	Range
6PPD	0.43	0.45	ND–0.90	ND	-	ND	0.13	0.20	ND–0.41	0.17	0.28	ND–0.74	0.34	0.60	ND–1.64	0.22	0.46	ND–1.22
7PPD	ND	-	ND	ND	-	ND	ND	-	ND	0.07	0.19	ND–0.58	0.10	0.17	ND	ND	-	ND
8PPD	ND	-	ND	ND	-	ND	ND	-	ND	ND	-	ND	ND	-	ND	ND	-	ND
CPPD	ND	-	ND	ND	-	ND	ND	-	ND	ND	-	ND	ND	-	ND	ND	-	ND
DNPD	ND	-	ND	ND	-	ND	ND	-	ND	ND	-	ND	ND	-	ND	ND	-	ND
DPPD	ND	-	ND	ND	-	ND	ND	-	ND	ND	-	ND	ND	-	ND	ND	-	ND
IPPD	0.08	0.14	ND–0.24	0.03	0.06	ND–0.13	0.03	0.07	ND–0.16	0.13	0.19	ND–0.44	0.04	0.10	ND–0.26	0.09	0.18	ND–0.47
ΣPPDs	0.51	0.34	0.24–0.90	0.03	0.06	ND–0.13	0.15	0.19	ND–0.41	0.37	0.36	ND–1.01	0.48	0.73	ND–2.05	0.31	0.50	ND–1.22
445	ND	-	ND	ND	-	ND	ND	-	ND	ND	-	ND	ND	-	ND	ND	-	ND
2N	0.22	0.38	ND–0.65	0.1	0.15	ND–0.34	ND	-	ND	0.51	0.48	ND–1.71	0.09	0.19	ND–0.49	1.05	0.43	0.41–1.78
TMQ	1.03	0.42	0.56–1.35	3.23	3.38	ND–7.78	5.48	3.41	ND–10.58	9.82	3.46	5.29–16.52	4.73	4.56	ND–10.18	7.76	1.97	4.68–10.77
ΣNON-PPDs	1.25	0.65	0.56–1.84	3.33	3.35	ND–7.78	5.48	3.41	ND–10.58	10.34	3.61	5.71–17.09	4.83	4.68	ND–10.67	8.82	2.13	5.41–11.87
HMMM	ND	-	ND	1.12	1.60	ND–3.47	2.79	2.73	ND–6.63	ND	-	ND	ND	-	ND	ND	-	ND
6PPD-Q	ND	-	ND	0.05	0.11	ND–0.25	ND	-	ND	0.17	0.34	ND–0.84	ND	-	ND	0.22	0.23	ND–0.55
IPPD-Q	0.05	0.04	ND–0.08	0.13	0.28	ND–0.62	0.13	0.32	ND–0.78	0.16	0.33	ND–0.89	ND	-	ND	0.06	0.15	ND–0.39
ΣPPD-Qs	0.05	0.04	ND–0.08	0.17	0.27	ND–0.62	0.13	0.32	ND–0.78	0.34	0.41	ND–0.89	ND	-	ND	0.27	0.34	ND–0.93
4-NOH	ND	-	ND	ND	-	ND	ND	-	ND	ND	-	ND	ND	-	ND	0.31	0.07	0.21–0.40
4OH	0.79	0.20	0.60–1.00	0.51	0.56	ND–1.35	0.86	1.23	ND–3.34	3.12	1.85	1.64–7.14	1.34	1.62	ND–4.13	3.38	1.66	1.28–5.74
ΣHPs	0.79	0.20	0.60–1.00	0.51	0.56	ND–1.35	0.86	1.23	ND–3.34	3.16	1.83	1.64–7.14	1.34	1.62	ND–4.13	3.70	1.72	1.49–6.10
ΣRARTPs	2.59	0.88	1.62–3.33	5.16	4.88	0.98–13.36	9.41	4.49	5.10–17.48	14.20	4.72	8.63–21.77	6.64	6.21	0.62–15.08	13.10	3.26	8.26–16.95

**Table 4:** Comparison of 6PPD, IPPD, and 6PPD-Q in various water samples of Kaifeng and other representative areas (ng·L^−^^1^).

Study Area	Sample Type	6PPD	IPPD	6PPD-Q	Reference
Kaifeng, China	surface water	ND–1.64 (0.20 ± 0.38)	ND–0.46 (0.07 ± 0.14)	ND–0.84 (0.09 ± 0.21)	This study
Liuxi River, China	surface water	ND	ND–2.14 (avg: 0.31)	ND–1.42	[18]
Don River, Canada	surface water during storm events	ND	-	110–540	[37]
Highland Creek, Canada		ND	-	210–720	
Seattle, U.S.	runoff	-	-	0.8–19000	[2]
Los Angeles, U.S.	runoff	-	-	4100–6100	
San Francisco, U.S.	runoff	-	-	1000–3500	
Saskatoon, Canadian	runoff	86–1400 (avg: 593)	-	-	[14]
Seattle, U.S.	watersheds	-	-	<0.3–3200	[2]
Don River, Toronto, Canada	during rain events and a snow melt event	-	-	300–2300	[38]
Nanaimo, BC, Canada	stormwater	-	-	50–5500	[39]
City of Leipzig, Germany	wastewater treatment plants	ND–105	-	-	[5]

**Table 5:** Concentrations of some RARTPs in Chinese riverine waters [40].

	Songhua–Liao River Basin (*n* = 10)	Huang River Basin (*n* = 6)	Hai River Basin (*n* = 5)	Yangtze River Basin (*n* = 13)	Pearl River Basin (*n* = 5)	Tarim River Basin (*n* = 2)	Brahmaputra River Basin (*n* = 2)	Southeast River Basin (*n* = 2)
6PPD	0.11–3.67 (avg: 1.09)	0.35–1.17 (avg: 0.66)	0.17–1.63 (avg: 0.67)	0.11–4.15 (avg: 1.23)	0.07–15.3 (avg: 3.75)	0.19–7.38 (avg: 3.78)	0.36–0.51 (avg: 0.43)	0.25–0.46 (avg: 0.36)
7PPD	-	-	-	-	-	-	-	-
8PPD	-	-	-	-	-	-	-	-
CPPD	0.56–55.6 (avg: 8.43)	0.74–6.16 (avg: 2.14)	0.99–5.22 (avg: 3.31)	0.16–7.00 (avg: 2.80)	1.90–11.3 (avg: 5.46)	2.00–7.61 (avg: 4.81)	1.08–1.92 (avg: 1.50)	1.03–1.30 (avg: 1.16)
DNPD	0.31–16.6 (avg: 5.21)	0.30–29.3 (avg: 12.2)	1.26–11.0 (avg: 4.30)	0.28–17.9 (avg: 5.27)	1.70–9.00 (avg: 4.68)	1.50–6.06 (avg: 3.78)	3.97–14.1 (avg: 9.05)	1.32–3.48 (avg: 2.44)
DPPD	0.79–5.42 (avg: 2.28)	0.53–1.37 (avg: 0.96)	0.99–48.4 (avg: 10.9)	0.36–3.94 (avg: 1.39)	0.87–5.65 (avg: 2.27)	2.40–8.05 (avg: 5.23)	0.85–1.15 (avg: 1.00)	0.89–3.20 (avg: 2.09)
IPPD	2.73–106 (avg: 25.0)	2.52–19.3 (avg: 9.45)	4.67–19.0 (avg: 11.8)	1.85–68.2 (avg: 23.9)	2.83–165 (avg: 44.4)	7.15–182 (avg: 94.5)	2.91–5.60 (avg: 4.25)	2.93–2.96 (avg: 2.95)

## Data Availability

The original contributions presented in this study are included in the article/Supplementary Material. Further inquiries can be directed to the corresponding author.

## Supplementary Materials

The following supporting information can be downloaded at: https://www.mdpi.com/article/10.3390/toxics14060521/s1, Figure S1: Sensitivity analysis results on surface water RARTP chronic daily intake assessment for population groups: (A) ingestion via drinking; (B) dermal absorption via bathing; (C) unintentional water ingestion via swimming; (D) dermal absorption via swimming; Table S1: Information of the sampling sites; Table S2: Basic information of the target analytes, reagents, and standards; Table S3: The calibration curves, IDL, IQL, and relative standard deviations (RSDs) of RARTPs; Table S4: Variables and parameters for calculating chronic daily intake; Table S5: Cumulative probability evaluation results of *TCDI* under Scenario 3 based on Monte Carlo simulation of surface water RARTPs for population groups in the study area (ng·(kg·d)^−1^); Text S1: Supporting Methodology (Potential ecological risk assessment and Estimated chronic daily intakes) [71].

## Abbreviations

RARTPs	Rubber additives and relevant transformation products
PPDs	*p*-Phenylenediamine antioxidants
PPD-Qs	*p*-Phenylenediamine quinones
HPs	Hydrolysis products
N-PPDs	Non-*p*-Phenylenediamine Antioxidants
BA	Binding agent
6PPD	*N*-(1,3-Dimethylbutyl)-*N*′-phenyl-*p*-phenylenediamine
7PPD	*N*,*N*′-bis(1,4-dimethylpentyl)-*p*-phenylenediamine
8PPD	*N*-(1-Methylheptyl)-*N*′-phenyl-*p*-phenylenediamine
CPPD	*N*-Cyclohexyl-*N*′-phenyl-*p*-phenylenediamine
DNPD	*N*,*N*′-Bis(2-naphthyl)-*p*-phenylenediamine
DPPD	*N*,*N*-Diphenyl-*p*-phenylenediamine
IPPD	*N*-isopropyl-*N*′-phenyl-*p*-phenylenediamine
6PPD-Q	*N*-(1,3-Dimethylbutyl)-*N*′-phenyl-*p*-phenylenediamine quinone
IPPD-Q	*N*-isopropyl-*N*′-phenyl-*p*-phenylenediamine quinone
4-NOH	4-Nitrosodiphenylamine
4OH	4-Hydroxydiphenylamine
445	4,4′-Bis(*α*,*α*-dimethylbenzyl) diphenylamine
2N	4-Aminodiphenylamine
TMQ	1,2-Dihydro-2,2,4-trimethylquinoline
HMMM	2,4,6-Tris[bis(methoxymethyl)amino]-1,3,5-triazine
6PPD-Q-d_5_	*N*-(1,3-Dimethylbutyl)-*N*′-phenyl-*p*-phenylenediamine quinone-d_5_
BP-d_10_	Benzophenone-d_10_
*TCDI*	Total chronic daily intake
*CDI*s	Chronic daily intakes
*RQ*	Risk quotient
*LC*_50_	Median lethal concentration
YR	Yellow River
NAR	Northern area river
NAL	Northern area lake
UAR	Urban area river
UAL	Urban area lake
SAR	Southern area river
TN	Total nitrogen
TOC	Total organic carbon
TC	Total carbon
IC	Inorganic carbon
HLB	Hydrophilic–lipophilic balance
*CDI*_ing-drink_	Chronic daily intake through drinking water ingestion
*CDI*_derm-bathe_	Chronic daily intake through dermal absorption during bathing
*CDI*_ing-swim_	Chronic daily intake through incidental water ingestion during swimming
*CDI*_derm-swim_	Chronic daily intake through dermal absorption during swimming
*IR*_swim_	Unintentional water ingestion rate during swimming
*EF*_swim_	Exposure frequency for swimming
*EF*_drink/bathe_	Exposure frequency for drinking
*DE*	Daily excretion
*SA*	Exposed skin area
*BW*	Average body weight
