# Supplementary material for: Occurrence, Ecological Risk, and Human Exposure of Rubber Additives and Transformation Products in Surface Waters of Kaifeng, China
Source: Toxics. 2026 Jun 15;14(6):521. doi: 10.3390/toxics14060521 (PMC13308114; doi:10.3390/toxics14060521)
Supplement: Supplementary file 1 [file toxics-14-00521-s001.zip › toxics-4322388-supplementary.pdf]

Occurrence, ecological risk, and human exposure of rubber additives and transformation products in surface waters of Kaifeng, China

Xing Chen [1,*,a], Chenyang Sun [2,a], Lingnan Du [1], Xinding Yao [1], Haifeng Wang [1], Zongwu Wang [1], Jiapu Ji [1], and Jinting Huang [3,*]

[1] School of Environmental Engineering, Yellow River Conservancy Technical University, Henan Engineering Technology Research Center of Green Coating Materials, Kaifeng Engineering Technology Research Center of Aquatic Environmental Pollution Monitoring, Kaifeng Key Laboratory of Food Composition and Quality Assessment, Kaifeng, Henan 475004, P.R. China; nikinew@126.com (L.D.); yaoxinding126@126.com (X.Y.); wanghaifeng@yrcti.edu.cn (H.W.); kf0986@163.com (Z.W.); jjp9122005@163.com (J.J.)

[2] Research and Development Center for Watershed Environmental Eco-Engineering, Advanced Institute of Natural Sciences, Beijing Normal University, Zhuhai, Guangdong 519087, P.R. China; 15893863095@163.com (C.S.)

[3] College of Surveying and Mapping Engineering, Yellow River Conservancy Technical University, Kaifeng, Henan 475004, China

---

[1] These authors contributed equally to this work.

[*] Corresponding author

E-mail: xchen0528@163.com, Tel.: +86-189-1689-0892 (X.C.); Jenkins1204@126.com, Tel.: +86-178-3922-1547 (J.H.)

S1 Supporting Methodology

Potential ecological risk assessment. Potential ecological risk assessment was performed according to the technical guidelines of the European Commission [1]. The risk quotient (*RQ*) was calculated using Eq S1:

$$RQ = MEC / PNEC_{water} \quad \text{S1}$$

where *MEC* was the measured concentration of the RARTPs in the surface water sample, and $PNEC_{water}$ was predicted to have no effect on the concentration. The *PNEC*s of 13 compounds were obtained from previous literature [2] (Table S2). The *PNEC* values of RARTPs were obtained by dividing their acute toxicity data by an assessment factor of 1000 [1]. The potential ecological risk of each sample site was classified according to the *RQ* values: high risk ($RQ \geq 1$), median risk ($0.1 \leq RQ < 1$), low risk ($0.01 \leq RQ < 0.1$), and minimal risk ($RQ < 0.01$) [3].

Estimated chronic daily intakes (*CDIs*). The chronic daily intake (*CDI*) of RARTPs by surface water intake was calculated according to the following equations [4]:

$$CDI_{ing\text{-}drink} = \frac{C_w \times IR_{drink} \times EF_{drink} \times ED}{BW \times AT} \quad \text{S2}$$

$$CDI_{derm\text{-}bathe} = \frac{C_w \times SA \times EF_{bathe} \times ET_{bathe} \times ED \times PC \times CF}{BW \times AT} \quad \text{S3}$$

$$CDI_{ing\text{-}swim} = \frac{C_w \times IR_{swim} \times EF_{swim} \times SF \times ED}{BW \times AT} \quad \text{S4}$$

$$CDI_{derm\text{-}swim} = \frac{C_w \times SA \times ET_{swim} \times EF_{swim} \times ED \times PC \times CF}{BW \times AT} \quad \text{S5}$$

Where, C_w is the content of the selected compounds in surface water, ng·L^{-1}; IR_{drink}: ingestion rate for drinking, L·d^{-1}; IR_{swim}: unintentional water ingestion rate during swimming, L·d^{-1}; $EF_{drink/bathe}$: exposure frequency for drinking, d·a^{-1}; EF_{swim}: exposure frequency for swimming, month·a^{-1}; *SF*, swimming frequency, event·month^{-1}; *ED*: exposure duration, a; *AT*: averaging time, AT=*ED*×365 d; *SA*: exposed skin area, cm^2; *BW*: average body weight, kg; ET_{bathe}: the time of bathing, min·d^{-1}; *PC*: the permeability coefficient for a chemical permeating through skin via water exposure in cm·h^{-1}, taking 0.001 cm·h^{-1} [5]; *CF*: the unit conversion factor for reconciling the C_w with the *SA* and *PC*, taking 1 L·cm^{-3}. Detailed information is listed in Table S4.

S2 Supplementary Data Tables

Table S1. Information of the sampling sites.

**Yellow River (YR)**		**Northern area lakes (NAL)**		**Urban area rivers (UAR)**		**Urban area lakes (NAL)**		**Southern area rivers (SAR)**	
**Sample sites**	**coordinates**	**Sample sites**	**coordinates**	**Sample sites**	**coordinates**	**Sample sites**	**coordinates**	**Sample sites**	**coordinates**
YR1 [a]	114.256890<br>34.882351	NAL1 [a]	114.349152<br>34.811464	UAR1	114.324703<br>34.824358	UAL1	114.272039<br>34.797667	SAR1	114.397214<br>34.756858
YR2 [a]	114.337613,<br>34.895862	NAL2	114.286610<br>34.871150	UAR2	114.325579<br>34.802106	UAL2	114.278395<br>34.811493	SAR2	114.414700<br>34.751526
YR3 [a]	114.354872,<br>34.908672	NAL3	114.344741<br>34.859567	UAR3	114.325097<br>34.785696	UAL3	114.282637<br>34.823868	SAR3	114.432731<br>34.735078
**Northern area rivers (NAR)**		NAL4	114.270593<br>34.846549	UAR4	114.343555<br>34.782528	UAL4	114.332278<br>34.792753	SAR4	114.446221<br>34.724932
NAR1 [a]	114.256890<br>34.884611	NAL5	114.270455<br>34.847717	UAR5	114.362405<br>34.778669	UAL5	114.341168<br>34.807555	SAR5	114.4146935<br>34.728084
NAR2	114.286703<br>34.871533	NAL6	114.255486<br>34.864179	UAR6	114.293902<br>34.761166	UAL6	114.340813<br>34.807510	SAR6	114.415798<br>34.728262
NAR3	114.344604<br>34.859608	-	-	UAR7	114.334463<br>34.796577	UAL7	114.366396<br>34.813984	SAR7	114.322880<br>114.322880
NAR4	114.256268<br>34.863083	-	-	UAR8	114.334818<br>34.801102	-	-	-	-
NAR5	114.260039<br>34.858661	-	-	UAR9	114.402234<br>34.775760	-	-	-	-

[a] Drinking water source point

Table S2. Basic information of the target analytes, reagents, and standards.

Abbreviation	Analyte	CAS	Molecular Formula	Molecular Weight	Reagent grades	Supplier	*PNEC*[f,g] [6,7]
6PPD	*N*-(1,3-Dimethylbutyl)-*N'*-phenyl-*p*-phenylenediamine	793-24-8	$C_{18}H_{24}N_2$	268.19	GC grade	*a*	45 ng·L^{-1}
7PPD	*N*, *N'*-bis(1,4-dimethylpentyl)-*p*-phenylenediamine	3081-01-4	$C_{19}H_{26}N_2$	282.42	GC grade	*b*	43 ng·L^{-1}
8PPD	*N*-(1-Methylheptyl)-*N'*- phenyl-*p*-phenylenediamine	15233-47-3	$C_{20}H_{28}N_2$	296.45	GC grade	*c*	16 ng·L^{-1}
CPPD	*N*-Cyclohexyl-*N'*-phenyl-*p*-phenylenediamine	101-87-1	$C_{18}H_{22}N_2$	266.18	GC grade	*b*	36 ng·L^{-1}
DNPD	*N,N'*-Bis(2-naphthyl)-*p*-phenylenediamine	93-46-9	$C_{26}H_{20}N_2$	360.16	GC grade	*c*	0.62 ng·L^{-1}
DPPD	*N,N*-Diphenyl-*p*-phenylenediamine	74-31-7	$C_{18}H_{16}N_2$	260.33	GC grade	*d*	29 ng·L^{-1}
IPPD	*N*-isopropyl-*N'*-phenyl-*p*-phenylenediamine	101-72-4	$C_{15}H_{18}N_2$	226.15	GC grade	*c*	140 ng·L^{-1}
6PPD-Q	*N*-(1,3-Dimethylbutyl)-*N'*-phenyl-*p*-phenylenediamine quinone	2754428-18-5	$C_{18}H_{22}N_2O_2$	298.17	GC grade	*d*	0.095 ng·L^{-1}
IPPD-Q	*N*-isopropyl-*N'*-phenyl-*p*-phenylenediamine quinone	68054-73-9	$C_{15}H_{16}N_2O_2$	256.12	GC grade	*d*	1.26 ng·L^{-1}
4-NOH	4-Nitrosodiphenylamine	156-10-5	$C_{12}H_{10}N_2O$	198.22	GC grade	*c*	450 ng·L^{-1}
4OH	4-Hydroxydiphenylamine	122-37-2	$C_{12}H_{11}NO$	185.08	GC grade	*c*	-
445	4,4'-Bis(*α*,*α*-dimethylbenzyl) diphenylamine	10081-67-1	$C_{30}H_{31}N$	405.57	GC grade	*c*	2.4 ng·L^{-1}
2N	4-Aminodiphenylamine	101-54-2	$C_{12}H_{12}N_2$	184.24	GC grade	*b*	1000 ng·L^{-1}
TMQ	1,2-Dihydro-2,2,4-trimethylquinoline	147-47-7	$C_{12}H_{15}N$	173.12	GC grade	*b*	-
HMMM	2,4,6-Tris[bis(methoxymethyl)amino]-1,3,5-triazine	3089-11-0	$C_{15}H_{30}N_6O_6$	390.44	GC grade	*c*	17 ng·L^{-1}
6PPD-Q-d_5	*N*-(1,3-Dimethylbutyl)-*N'*-phenyl-*p*-phenylenediamine quinone-d_5	2750119-14-1	$C_{18}H_{17}D_5N_2O_2$	303.4	GC grade	*b*	-
BP-d_{10}	Benzophenone-d_{10}	22583-75-1	$C_{13}D_{10}O$	192.28	GC grade	*d*	-
-	Sodium chloride	7647-14-5	NaCl	-	Analytical grade	*e*	-
-	Methanol	67-56-1	CH_3OH	-	GC grade	*e*	-
-	Acetonitrile	75-05-8	CH_3CN	-	GC grade	*e*	-

[a] AccuStandard, Inc. (New Haven, CT, USA); [b] Cato Research Chemicals Inc. (Guangzhou, Guangdong, China); [c] Tokyo Chemical Industry (Tokyo, Japan); [d] J&K Scientific (Beijing, China); [e] ANPEL Laboratory Technologies (Shanghai) Inc., China; [f] ECHA. (https://echa.europa.eu/de/information-on-chemicals/registered-substances.); [g] NORMAN Database System. (https://www.norman-network.com/nds/common/).

Table S3. The calibration curves, IDL, IQL, and relative standard deviations (RSD) of RARTPs.

Compound Name	Calibration curves		IDL ($\mu g \cdot L^{-1}$)	IQL ($\mu g \cdot L^{-1}$)	RSD (%)
	Concentration ($\mu g \cdot L^{-1}$)	Regression coefficients (R^2)			
6PPD	0.05-100	0.999	0.05	0.1	6.49
7PPD	0.02-100	0.997	0.02	0.05	12.09
8PPD	0.05-100	0.992	0.05	0.1	9.30
CPPD	0.05-100	0.999	0.05	0.1	12.40
DNPD	0.5-100	0.999	0.5	1	12.70
DPPD	0.2-100	0.999	0.2	0.5	12.90
IPPD	0.02-100	0.996	0.02	0.05	9.04
6PPD-Q	0.05-100	0.995	0.05	0.1	13.51
IPPD-Q	0.2-100	0.999	0.2	0.5	13.20
445	0.05-100	0.996	0.05	0.1	13.32
2N	0.1-100	0.999	0.1	0.2	14.30
4-NOH	0.02-100	0.999	0.02	0.05	12.67
TMQ	0.2-100	0.995	0.5	1	10.20
HMMM	0.02-100	0.995	0.02	0.05	8.68

Note: IDL means the instrument detection limit;
IQL means the instrument quantification limit.

Table S4. Variables and parameters for calculate chronic daily intake.

		Children [8-11]					Adults [12,13]			
	Age	0~5	6~<9	9~<12	12~<15	15~<18	18~44	45~59	60~79	≥80
IR_{drink} (mL·d^{-1})	Male	858	1184	1473	1403	1491	2376	2471	2358	2041
	Female	869	1253	1549	1386	1463	2219	2091	1894	1556
$IR_{swallowed}$ (mL·event^{-1})	Male	40.5	40.5	40.5	40.5	40.5	27.0	27.0	27.0	-
	Female	40.5	40.5	40.5	40.5	40.5	40.5	40.5	40.5	40.5
SF (event·month^{-1})	Male	4.3	4.3	4.3	3.3	4.3	8	8	8	8
	Female	4.3	4.3	4.3	3.3	4.3	4	4	4	4
ED (a)		6					30			
EF_{drink}/EF_{bathe} (d·a^{-1})		365					365			
EF_{swim} (month·a^{-1})		3					3			
AT (d)		18×365					Male: 71.84×365<br>Female: 77.59×365			
BW (kg)	Male	15.8	27.1	38.1	50.2	58.6	69.0	69.3	65.8	62.3
	Female	15.3	25.8	35.7	46.4	51.3	57.1	60.7	59.4	54.3
ET_{bathe} (min·d^{-1})	Male	7	9	11	11	10	8	8	7	7
	Female	7	9	11	13	14	10	8	7	7
ET_{swim} (min·month^{-1})	Male	89	144	224	236	252	218	179	112	-
	Female	84	153	207	194	183	186	217	277	105
SA (m^2)	Male	0.68	1.03	1.29	1.59	1.73	1.8	1.8	1.8	1.7
	Female	0.66	0.97	1.25	1.46	1.59	1.6	1.6	1.6	1.6

Table S5. Cumulative probability evaluation results of *TCDI* under Scenario 3 based on Monte Carlo simulation of surface water RARTPs for population groups in the study area (ng·(kg·d)$^{-1}$).

	**Boy**	**Male adult**	**Girl**	**Female adult**
5th	8.83E-02	7.13E-02	9.91E-02	6.79E-02
10th	9.87E-02	8.00E-02	1.10E-01	7.59E-02
25th	1.18E-01	9.60E-02	1.31E-01	9.08E-02
50th	1.42E-01	1.16E-01	1.57E-01	1.10E-01
75th	1.69E-01	1.40E-01	1.85E-01	1.31E-01
90th	1.97E-01	1.64E-01	2.13E-01	1.52E-01
95th	2.14E-01	1.79E-01	2.30E-01	1.65E-01
Mean	1.46E-01	1.20E-01	1.60E-01	1.12E-01
Minimum	1.42E-01	1.16E-01	1.57E-01	1.10E-01
Maximum	6.29E-02	4.99E-02	7.20E-02	4.82E-02
Range Width	4.01E-01	3.56E-01	3.98E-01	3.12E-01
Mean Std. Error	3.38E-01	3.06E-01	3.26E-01	2.64E-01

S3 Supplementary Figures

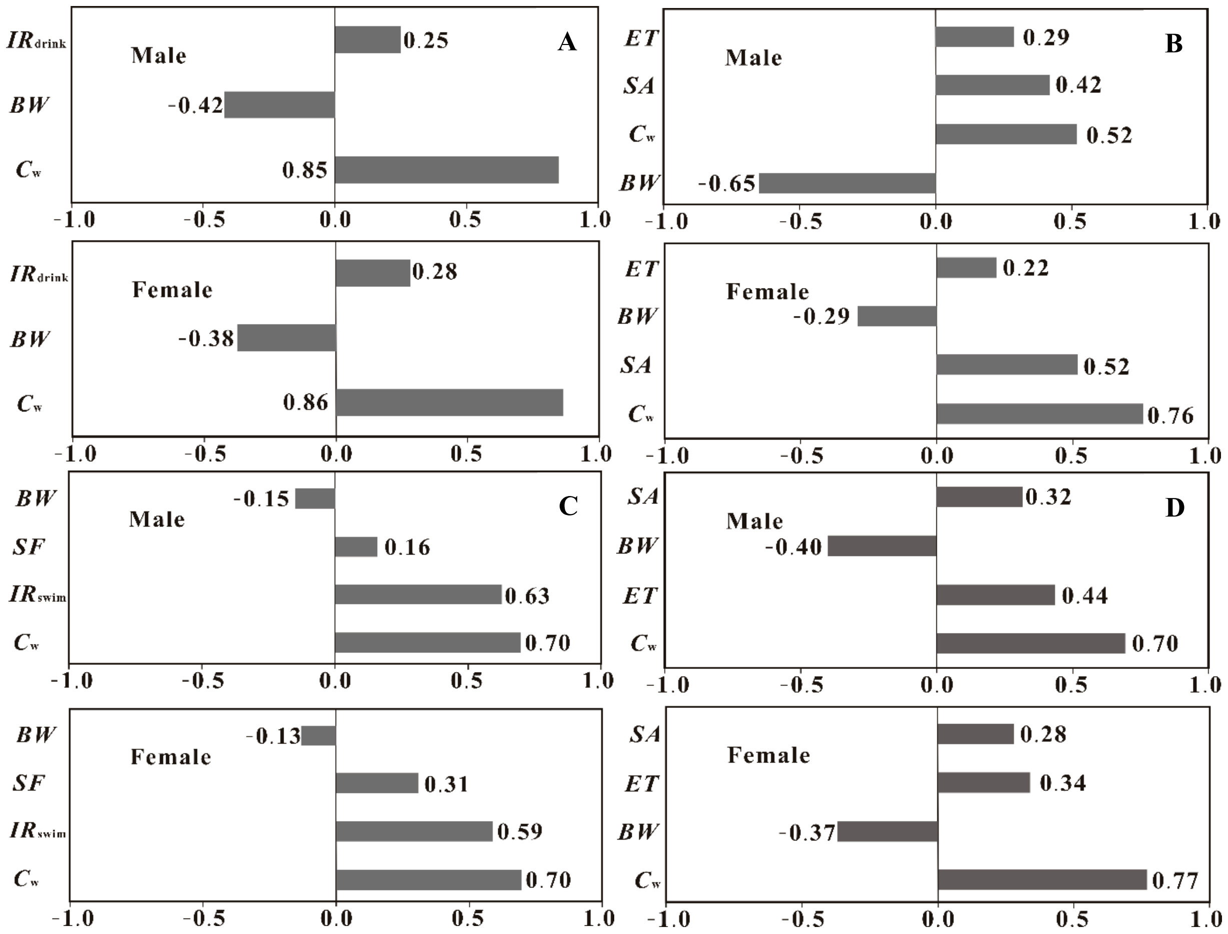

Figure S1. Sensitivity analysis results on surface water RARTPs chronic daily intake assessment for population groups, (A) ingestion via drinking; (B) dermal absorption via bathing; (C) unintentional water ingestion via swimming; (D) dermal absorption via swimming.

References

1 European Commission (EC). Technical Guidance Document in support of Commission Directive 93/67/EEC on risk assessment for new notified substances and Commission Regulation (EC) No1488/94 on risk assessment for existing substances Part II, Office for official publications of the European communities, Italy. **2003**.

2 Zhang, H.-Y.; Huang, Z.; Liu, Y.-H.; Hu, L.-X.; He, L.-Y.; Liu, Y.-S.; Zhao, J.-L.; Ying, G.-G. Occurrence and risks of 23 tire additives and their transformation products in an urban water system. *Environ. Int*. **2023**, 171, 107715.

3 Hernando, M.-D.; Mezcua, M.; Fernandez-Alba, A.-R.; Barcelo, D. Environmental risk assessment of pharmaceutical residues in wastewater effluents, surface waters and sediments. *Talanta*. **2006**, 69(2), 334-342.

4 Liu, A.-X.; Lang, Y.-H.; Xue, L.-D.; Liu, J. Ecological risk analysis of polycyclic aromatic hydrocarbons (PAHs) in surface sediments from Laizhou Bay. *Environ. Monit. Assess*. **2009**, 159, 429-436.

5 Zhang, X.-L., Zhang, Z.-H., Xu, Z.-J., Gu, D.-Q., Zheng, W. Landscape pattern change and its cumulative environmental effects of coastal wetlands in southern Laizhou Bay. *Chinese Journal of Ecology*. **2009**, 28, 2437-2443.

6 Tian, Z.; Zhao, H.; Peter, K.; Gonzalez, M.; Wetzel, J.; Wu, C.; Hu, X.; Prat, J.; Mudrock, E.; Hettinger, R.; Cortina, A.; Biswas, R.; Kock, F.; Soong, R.; Jenne, A.; Du, B.; Hou, F.; He, H.; Lundeen, R.; Kolodziej, E. A ubiquitous tire rubber derived chemical induces acute mortality in coho salmon. *Science*. **2020**, 371. https://doi. org/10.1126/science.abd6951.

7 Zhang, H.-Y.; Huang, Z.; Liu, Y.-H.; Hu, L.-X.; He, L.-Y.; Liu, Y.-S.; Zhao, J.-L.; Ying, G.-G. Occurrence and risks of 23 tire additives and their transformation products in an urban water system. *Environ. Int*. **2023**, 171, 107715.

8 Wang, B.-B., Duan, X.-L. Exposure factors handbook of Chinese population (0-5 years). China Environmental Science Pres. **2016**. (In Chinese)

9 Zhao, X.-G, Duan, X.-L. Exposure factors handbook of Chinese population (6-17 years). China Environmental Science Pres. **2016**. (In Chinese)

10 Zhao, X.-G., Duan, X.-L. Report of environmental exposure related activity patterns research of Chinese population (Children). China Environmental Science Pres. **2016**. (In Chinese)

11 Liu, Q.-C, Hu, L.-M, Sun, J. Analysis of Exposure Parameters for Child Swimmers in Indoor Swimming Pools during Summer in Shenzhen. *Applied. Prev. Med*. **2025**, 31(05), 461-464+470. (In Chinese)

12 Duan, X.-L. Exposure factors handbook of Chinese population. China Environmental Science Pres. **2013**. (In Chinese)

13 He, X.-T; Liu, Q.-C; Sun, J. Investigation of exposure parameters among 215 adult swimmers in summer indoor swimming pool in Baoan district, Shenzhen in 2023. *Modern Disease Control and Prevention*. **2023**, 34(12), 945-949. DOI:10.13515/j.cnki.hnjpm.1006-8414.2023.12.016. (In Chinese)
